# Mediators of Inflammation in Myeloproliferative Neoplasms: State of the Art

**DOI:** 10.1155/2015/964613

**Published:** 2015-11-23

**Authors:** Sylvie Hermouet, Hans C. Hasselbalch, Vladan Čokić

**Affiliations:** ^1^Inserm UMR892/CNRS UMR6299, Centre de Recherche en Cancérologie Nantes-Angers, Institut de Recherche en Santé, Université de Nantes, 44007 Nantes, France; ^2^Laboratoire d'Hématologie, Centre Hospitalier Universitaire (CHU), 44093 Nantes, France; ^3^Department of Hematology, Roskilde Hospital, University of Copenhagen, Copenhagen, Denmark; ^4^Institute for Medical Research, Laboratory of Experimental Hematology, University of Belgrade, 11129 Belgrade, Serbia

Myeloproliferative neoplasms (MPNs) are a heterogeneous group of chronic clonal diseases which are characterized by the excessive production of mature cells of one or more of the myeloid lineages. The three main MPN subtypes are very different diseases of distinct clinical presentation, evolution, and variable severity, ranging from mild (essential thrombocythemia (ET)) to moderate (polycythemia vera (PV)) or severe (primary myelofibrosis (PMF)). Recent advances in the biology of MPNs have greatly facilitated the molecular diagnosis of MPNs, since a large majority of MPN patients present with mutation in one of three genes:* JAK2, CALR, *and, more rarely,* MPL*. However, important questions remain regarding the role of these mutations in the pathogenesis of MPNs. While it is established that mutations in the* MPL* and* JAK2* genes result in increased activation of the JAK2/STAT5, STAT3, and STAT1 pathways and hypersensitivity of myeloid progenitors to hematopoietic cytokines, the consequences of* CALR* mutations are not fully understood. Moreover, the reasons why patients present with ET, PV, or PMF when the* JAK2*-V617F mutation is found in all subtypes of MPNs, and the* CALR* and* MPL* mutations are present in ET as well as in PMF, are still not elucidated.

It has long been known that MPN patients present with elevated levels of numerous inflammatory cytokines in blood and bone marrow. Over time several groups established links between inflammatory cytokine levels and MPN phenotype, clinical symptoms, and certain complications. More recently, JAK inhibitors were shown to efficiently decrease the production and signaling of major inflammatory cytokines, leading to significant reduction of spleen size and other invalidating inflammation-linked clinical symptoms in PMF patients while the* JAK2*-mutated MPN clone remained unaffected, as assessed by quantification of the* JAK2* mutant allelic burden. Thus a better knowledge of the causes and molecular mechanisms that underlie inflammation in MPNs could improve understanding of the pathogenesis of MPNs and the treatments proposed to MPN patients.

To help achieve this aim, we invited authors who could shed light on the contribution of inflammation (cytokines and other inflammation markers) to mutation acquisition, clone proliferation, biological parameters, clinical presentation, disease evolution, and response to treatment in MPNs. Major investigators in the field thus described, analyzed, and summarized the biological and clinical findings accumulated in regard to inflammation in MPNs. Our hope is that integrating inflammation in MPN pathophysiology can provide the rationale for novel therapeutic strategies for MPNs that target inflammation in addition to the main mutations.

In the paper entitled “Circulating Cytokine Levels as Markers of Inflammation in Philadelphia Negative Myeloproliferative Neoplasms: Diagnostic and Prognostic Interest”, J. Mondet et al. discuss the importance of cytokines in the development of MPNs and, subsequently, the interest of determining the circulating levels of cytokines at the time of diagnosis. This extensive review presents the current knowledge on cytokine profiles in the different subtypes of MPNs and the role of altered cytokine expression in the pathogenesis of myelofibrosis. Phenotypic correlation, prognostic value of inflammation cytokines in MPNs, and the impact of JAK inhibitors on inflammation cytokine levels are discussed. The authors conclude that circulating cytokines levels could be useful diagnostic and prognostic markers in MPNs. They suggest that cytokine assays could be useful in monitoring treatment efficacy, since a significant cytokine reduction could serve as an indirect marker for therapeutic compliance and efficacy.

In their paper “Impact of Inflammation on Myeloproliferative Neoplasm Symptom Development”, H. L. Geyer et al. describe inflammation markers characteristic of MPNs, the upstream sources stimulating their development, their prevalence within the MPN population, and the role they play in symptom development. They present Patient Reported Outcome (PRO) tools designed for evaluating the potential associations between symptoms and inflammation. The authors report clear relationships between individual MPN symptoms (fatigue, abdominal complaints, microvascular symptoms, and constitutional symptoms) and inflammatory cytokines (interleukin- (IL-) 1, IL-6, IL-8, and tumor necrosis factor- (TNF-) *α*). Information is also provided on the role symptoms paradoxically play in cytokine production, as in the case of fatigue-driven sedentary lifestyles. H. L. Geyer et al. conclude that increased attention should be paid to how inflammation markers differ between MPN subtypes, change with disease progression, and relate to transformation and anticipate novel inflammation-targeting therapies high potential for symptomatic benefit.

The paper of G. Hoermann et al., entitled “Cytokine Regulation of Microenvironmental Cells in Myeloproliferative Neoplasms,” reviews the role of MPN-related oncogenes in cytokine expression and release by neoplastic cells and the modulation of microenvironmental cells by these cytokines. The authors describe common as well as distinct pathogenic mechanisms underlying the microenvironmental changes observed in the bone marrow and other organs of MPN patients. Targeting of the microenvironment and of related cytokines (or their receptors) as an attempt to improve therapies in MPNs is also discussed, as such therapies may enhance the efficacy and overcome resistance to established tyrosine kinase inhibitors treatment in MPN patients. For instance, the VEGF/VEGFR, HGF/c-MET, and SDF-1/CXCR4 axes are presented as potential targets in MPNs. The authors conclude that increasing knowledge of the leukemic stem cell- (LSC-) niche interactions will assist in the development of new improved treatment approaches in MPN patients.

In the paper entitled “Inflammation as a Keystone of Bone Marrow Stroma Alterations in Primary Myelofibrosis,” C. Desterke et al. discuss bone marrow stroma alterations in PMF, as evidenced by myelofibrosis, neoangiogenesis, and osteosclerosis, and the involvement of altered stromal cells in PMF pathogenesis. The authors propose that the stroma may be inflammatory-imprinted* in vivo* by clonal hematopoietic cells and then becomes “independent” of hematopoietic cell stimulation, rendering this inflammatory loop unbreakable without the association of stroma-targeted therapies to the current protocols.

M. E. Bjørn and H. C. Hasselbalch in “The Role of Reactive Oxygen Species in Myelofibrosis and Related Neoplasms” describe how reactive oxygen species (ROS) are involved in MPN disease initiation and progression throughout the biological continuum from early cancer stage (ET/PV) to advanced cancer stage (myelofibrosis, or MF). Excess ROS, oxidative stress, as a result of chronic inflammation with consequent double stranded DNA breaks in combination with a germline predisposition with impaired DNA repair might account for genetic susceptibility in a subset of individuals implying an increased risk of acquiring an MPN when suffering from chronic inflammatory diseases. The authors hypothesize that the excess production of ROS, by the malignant clone itself, in addition to carcinogenesis also provides an escape from innate and adaptive tumor-immune-surveillance, mainly by blocking interferon signaling. How targeting of ROS with N-Acetyl-Cysteine (NAC) has been a success in other inflammation-driven diseases and why NAC-treatment should be pursued in MPNs as well are discussed. Furthermore, the systemic excess production of ROS also provides a link between the MPNs and MPN-associated comorbidities, in particular the cardiovascular disease-burden.

The paper of J. S. Jutzi and H. L. Pahl, with its provocative title “The Hen or the Egg: Inflammatory Aspects of Murine MPN Models,” addresses the contribution of inflammation and other changes in the bone marrow niche in the genesis and maintenance of MPNs. They compare data obtained in gastrointestinal tumors with observations in MPN patients and models and describe novel murine MPN models that may be used to address fundamental questions regarding the role of inflammation in the pathogenesis of MPNs.

In the paper entitled “MPNs as Inflammatory Diseases: The Evidence, Consequences, and Perspectives,” H. C. Hasselbalch and M. E. Bjørn describe the evidence for considering the MPNs as inflammatory diseases, “A Human Inflammation Model of Cancer Development,” and the role of cytokines in disease initiation and progression. The consequences of this model are discussed, including the increased risk of second cancers and other inflammation-mediated diseases, emphasizing the urgent need for rethinking our therapeutic approach. Early intervention with interferon-alpha 2, which as monotherapy has been shown to induce minimal residual disease in a subset of PV patients, in combination with potent anti-inflammatory agents such as JAK-inhibitors and statins is foreseen as the most promising new treatment modality in the years to come.

The paper of S. Hermouet et al. titled “Pathogenesis of Myeloproliferative Neoplasms: Role and Mechanisms of Chronic Inflammation” discusses the role played by the* JAK2*,* CALR,* and* MPL* mutations in the pathogenesis of the different subtypes of MPNs. The authors review the different aspects of inflammation in MPNs, the main molecular mechanisms involved, and the role of specific somatic or germline genetic defects in the production of inflammatory cytokines. They present evidence that certain inflammatory cytokines present in excess in MPNs are produced independently from the* JAK2*-V617F mutation. The authors also discuss several inflammation markers that have been identified in MPNs as predictive markers independently of the* JAK2*-V617F mutation and drugs that already exist and target these markers. They also describe possible nongenetic causes of inflammation.

A. G. Fleischman, in her paper “Inflammation as a Driver of Clonal Evolution in Myeloproliferative Neoplasm,” provides evidence that the impact of chronic inflammation goes far beyond its role as a driver of constitutional symptoms. The author shows that inflammatory response to the neoplastic clone may be responsible for different pathologic aspects of MPNs and that the* JAK2*V617F-mutated progenitor cells are resistant to the suppressive action of certain inflammatory cytokines, which gives the neoplastic clone a selective advantage and justifies targeting inflammation as a logical therapeutic approach in MPNs.

Last but not least, M. Sevin et al. in “HSP90 and HSP70: Implication in Inflammation Processes and Therapeutic Approaches for Myeloproliferative Neoplasms” report that heat shock proteins (HSP) are key players during inflammation, through their chaperone activity. Notably, HSP90 stabilizes many oncogenes including JAK2 and also key components of the Nuclear Factor-kappa B (NF-*κ*B) signaling pathway, which plays critical roles in the inflammatory response. HSP70 represents another HSP to consider since it tightly regulates the NF-*κ*B pathways. As expected, several HSP90 inhibitors generated as anticancer agents allow the degradation of oncogenes and inhibit the inflammatory response. M. Sevin et al. also discuss the emergence of HSP inhibitors in new protocols designed for the therapy of MPNs.

Altogether, the present special issue highlights the different aspects and the importance of the contribution of inflammation cytokines as crucial regulators of the MPN clone and as mediators of clinical symptoms and complications. The special issue also describes the effects of current drugs or combinations of drugs acting on inflammation in MPNs and proposes new lines of research and novel therapeutic strategies targeting inflammation in MPNs.

